# Effects of motor-cognitive interaction based on dual-task gait analysis recognition in middle age to aging people with normal cognition and mild cognitive impairment

**DOI:** 10.3389/fnagi.2022.969822

**Published:** 2022-10-04

**Authors:** Yuxin Zheng, Shijuan Lang, Junjie Liang, Yongchun Jiang, Biyi Zhao, Hongxin Chen, Dongqing Huang, Qinyi Li, Huijin Liu, Shudi Chen, Anniwaer Yilifate, Fangqiu Xu, Haining Ou, Qiang Lin

**Affiliations:** ^1^Department of Rehabilitation, The Fifth Affiliated Hospital of Guangzhou Medical University, Guangzhou, China; ^2^Department of Rehabilitation, Fifth Clinical College, Guangzhou Medical University, Guangzhou, China; ^3^Health Science Center, Shenzhen University, Shenzhen, China; ^4^Department of Rehabilitation, Zhanjiang Central Hospital, Guangdong Medical University, Zhanjiang, China; ^5^Department of Physiology and Pathophysiology, School of Basic Medical Sciences, Capital Medical University, Beijing, China; ^6^Department of Clinical Medicine, Guangzhou Medical University, Guangzhou, China; ^7^Key Laboratory of Biological Targeting Diagnosis, Therapy and Rehabilitation of Guangdong Higher Education Institutes, The Fifth Affiliated Hospital of Guangzhou Medical University, Guangzhou, China

**Keywords:** gait analysis, MCI, dual-task, dual-task cost, rehabilitation

## Abstract

**Background:**

Mild cognitive impairment (MCI) is considered a transitional stage between cognitive normality and dementia among the elderly, and its associated risk of developing Alzheimer’s disease (AD) is 10–15 times higher than that of the general population. MCI is an important threshold for the prevention and control of AD, and intervention in the MCI stage may be the most effective strategy to delay the occurrence of AD.

**Materials and methods:**

In this study, 68 subjects who met the inclusion criteria were divided into an MCI group (38 subjects) and normal elderly (NE) group (30 subjects). Both groups underwent clinical function assessments (cognitive function, walking function, and activities of daily living) and dual-task three-dimensional gait analysis (walking motor task and walking calculation task). Spatial-temporal parameters were obtained and reduced by principal component analysis, and the key biomechanical indexes were selected. The dual-task cost (DTC) was calculated for intra-group (task factor) and inter-group (group factor) comparisons.

**Results:**

The results of the principal component analysis showed that the cadence parameter had the highest weight in all three walking tasks. In addition, there were significant differences in the cadence both walking motor task (WMT) vs. walking task (WT) and walking calculation task (WCT) vs. WT in the MCI group. The cadence in the NE group only showed a significant difference between WMT and WT. The only differences between the MCI group and NE group was DTC cadence in WCT, and no differences were found for cadence in any of the three walking tasks.

**Conclusion:**

The results show that dual tasks based on cognitive-motor gait analysis of DTC_cadence_ in MCI have potential value for application in early identification and provide theoretical support to improve the clinical diagnosis of MCI.

## Introduction

Mild cognitive impairment (MCI) is considered as the transitional stage from a normal cognitive state to dementia in the elderly and is a typical clinical manifestation of pre-dementia (Petersen, 2004). A cross-sectional survey of Chinese communities showed that the prevalence of MCI in those over 55 years old is 14.5%, which increases with age. The risk of Alzheimer’s disease (AD) in MCI patients is 10–15 times higher than that in the general population (Liu-Ambrose et al., 2008). Therefore, MCI is an important gateway for interventions and the prevention of AD. Interventions in the MCI stage may be the most effective strategy to delay the occurrence of AD.

However, MCI is still symptomatically diagnosed in clinic, and neuropsychological assessment is still important in diagnosis and research on MCI (Chipi et al., 2019). Therefore, the evaluation results are semi-quantitative data, which are greatly affected by subjective factors and have poor consistency and low sensitivity. It is challenging to evaluate and diagnose MCI patients accurately. Studies have shown that the motor ability of MCI patients is reduced to a certain extent, and the risk of falls is higher than that of normal elderly people (Schwenk et al., 2016; Tyrovolas et al., 2016).

As an A-level recommendation, the 2018 Chinese Guidelines for the Diagnosis and Treatment of Dementia and Cognitive Impairment in China pointed out that gait performance can help with early identification and prediction of the progress of MCI (Classification Types and Causes of Diseases, 2018). Gait analysis with a single task shows a decline in gait performance in patients with MCI, especially in walking velocity (Grande et al., 2019). However, slow walking velocity may also be associated with aging (Noce Kirkwood et al., 2018). Therefore, gait velocity may be a non-specific variable, and assessing walking performance with a single task may be unsatisfactory for accurately identifying MCI. Thus, dual-task gait analysis was developed and provides a new perspective for the identification of early MCI.

Dual-task walking is walking while performing another task that occupies cognitive resources, such as using mobile phones or talking. Compared with single-task walking, dual-task walking is more challenging, more common in daily life, and more conducive to the discovery of potential gait abnormalities in activities of daily living (ADL) (Liang et al., 2019; Oh, 2021). Imaging studies have also shown that gait and cognitive control share a brain network, especially in the cognitive and motor zones of the frontal cortex (Rosano et al., 2006; Annweiler et al., 2012; Montero-Odasso et al., 2017). Manuel Montero-Odasso et al. confirmed the reliability of dual-task quantitative gait analysis for MCI recognition by analyzing the changes of gait parameters in single-task and dual-task walking (Montero-Odasso et al., 2009; Bishnoi and Hernandez, 2021). In addition, the dual-task cost (DTC) introduced in dual-task gait analysis can be used to calculate the dual-task consumption of cognitive resources. Compared with the original data, it reduces the influence of specific factors of patients, such as height, weight, physical health status, and physical activity level. Thus, it can more accurately quantify the dual-task performance and shows great potential in identifying MCI.

Nevertheless, locating and identifying important biomechanical indicators of MCI among many parameters is an urgent problem to solve and has great significance for guiding the clinical evaluation and diagnosis of MCI. In recent years, principal component analysis (PCA) has been widely used in biomechanics research. PCA is a dimensionality reduction algorithm that can convert multiple indicators into a few unrelated principal components. Thus, fewer unrelated variables can be used to reflect the information conveyed by a large number of relevant raw data. Wakako et al. extracted 26 principal component vectors from many gait parameters by the PCA method and found that knee-angle variability and ankle-angle variability are important gait indicators for distinguishing between frail and non-frail older women (Tsuchida et al., 2022). Therefore, the goal of this study is to increase the understanding of spatio-temporal parameters of MCI and normal elderly participants by using PCA.

In addition, the following key problems still need to be solved in clinical and mechanism research on dual-task gait analysis for early diagnosis of MCI. First, the key to diagnosis is distinguishing MCI from middle age to elderly population. MCI is mainly manifested as a decline in the function of single or multiple cognitive fields, but normal middle-aged and elderly population also shows a decline in cognitive ability due to factors such as reduced hippocampal volume and adverse changes in cerebrovascular structure (Sun et al., 2022). Therefore, distinguishing MCI from normal aging-induced cognitive decline is a key issue in the clinical diagnosis of MCI (Naidu et al., 2019).

Another problem is that further clarification is needed in regard to the difference in MCI recognition effect between different types of dual tasks needs. There are many types of dual tasks, and the sensitivity of different cognitive tasks to MCI recognition is not clear (Hunter et al., 2018; Bishnoi and Hernandez, 2021). Therefore, selecting different types of dual tasks may affect the accurate recognition of MCI. Therefore, in this study, three-dimensional gait analysis was used to compare the biomechanical characteristics of MCI patients and middle-aged and elderly population during conventional walking and different dual tasks. PCA was then used to explore the sensitive indicators for the objective evaluation of MCI patients, which could provide an important basis for clinical applications.

## Materials and methods

### Participants

This study recruited MCI elderly subjects and healthy elderly subjects in the Huangpu District of Guangzhou City. The inclusion criteria for MCI subjects were an age of 55–85 years, the DSM-5 diagnostic criteria for MCI, Montreal cognitive assessment (MoCA) score < 24 (Julayanont et al., 2014), and ability to walk independently without gait aids (such as a walking stick or walker). The inclusion criteria of healthy subjects were an age of 55–85 years, MoCA score ≥ 24 (consistent with a normal cognitive level), no aphasia, no history of psychosis, and walking independently without gait aids (such as crutches or walkers). The exclusion criteria for both groups were language dysfunction, history of Parkinson’s disease, or any neurological disorder (such as stroke or epilepsy), musculoskeletal diseases, a history of knee or hip arthroplasty that affects gait performance, consumption of psychotropic drugs that affect sports performance, history of mental illness, congestive heart failure, deep venous thrombosis of the lower extremities, malignant progressive hypertension, respiratory failure, active liver disease, and liver or kidney dysfunction.

This study was approved by the Ethics Committee of the Fifth Affiliated Hospital of Guangzhou Medical University (No. KY01-2019-03-01) and has been registered in an international clinical trials registry (ChiCTR1800018832^1^). All participants signed informed consent forms.

### Study design

This study is a cross-sectional control study. The subjects with MCI were included in the experimental group (MCI group), and the healthy subjects were included in the normal elderly group (NE group) as control. Both groups received the same assessment, including standardized questionnaire collection of basic information, clinical function assessment (including cognitive function, gait, and ADL assessment), and three-dimensional gait analysis (single task and dual task). Clinical function assessment was conducted by two experienced therapists, and three-dimensional gait analysis was conducted by a professional gait analysis technician in the Gait Analysis Laboratory of the Fifth Affiliated Hospital of Guangzhou Medical University.

### Clinical function evaluation

#### Cognitive function assessment

MoCA was used to evaluate cognitive function. The scale consists of 11 items in eight cognitive fields, including attention and concentration, executive function, memory, language, visual-spatial skills, abstract thinking, calculation, and orientation. The total score was 30, and scores ≥ 26 are regarded as normal cognitive function (Nasreddine et al., 2005).

#### Gait assessment

A 10-meter walking test (10WMT) was used to evaluate the functional walking ability of subjects. The subjects were asked to walk three times on a 10-m footpath at their own comfortable speed. A therapist used a stopwatch to measure the completion time and calculated the average speed of the middle 6 m of the three tests (m/s).

The Timed Up and Go test (TUGT) (Liang et al., 2019) is commonly used to screen the risk of fall for hospitalized patients and elderly people and can also be used as a monitoring tool for cognitive ability. The subject stood up from an armchair, which was about 46 cm high, crossed marked line 3 m away at a comfortable speed, turned back to the chair, and sat down while being protected and guided by an experienced therapist throughout the test. The therapist used a stopwatch to record the completion time and calculated the average time of the three tests. Times over 13.5 s were considered to indicate increased risk of falls (Barry et al., 2014).

#### Activity of daily living assessment

The Modified Barthel Index (MBI) was used to evaluate the ADL of subjects and includes 10 items: eating, dressing, toilet use, bathing, personal hygiene, bed/chair transfer, walking up and down stairs, defecation, and bladder control. Each activity can be divided into grades 1–5 (5 points) (Kaambwa et al., 2021). A higher grade indicates greater independence. A score of 21–60 means high dependence and a great need for help, 61–90 indicates moderate dependence and a need for help, 91–99 indicates mild dependence and basic self-care capability, and100 indicates complete independence and self-care capability. The total scores of the subjects were included in the data statistics.

### Three-dimensional gait analysis

A three-dimensional gait analysis system (Italy BTS SMART DX infrared motion capture and analysis system) was used to collect the motion trajectory of fluorescent markers on the subjects. Twenty-two markers were placed on the subjects, and three-dimensional spatial and temporal data were obtained through six high-precision infrared cameras while the subjects walked.

#### Subject preparation

The subjects wore tight underwear and with the neck, shoulder, waist, and lower limbs fully exposed. The height, weight, bilateral ankle width, bilateral knee-joint diameters, pelvic width, bilateral pelvic depth, and bilateral leg lengths were measured and recorded. According to the Davis Heel Protocol, 22 fluorescent markers were attached to the subject. Three markers were located on the trunk (seventh cervical vertebra, shoulders on both sides), 3 markers were located on the pelvis (bilateral anterior superior iliac spine and ankle joints), 6 markers were located on the thigh (bilateral femoral trochanter, femoral condyle and midpoint of femoral trochanter and ipsilateral femoral condyle), 6 markers were located on the calf (bilateral fibula head, lateral ankle joint and midpoint of ipsilateral fibula head and lateral ankle joint), and 4 markers were located on the foot (fifth metatarsal head and bilateral heels) (Ou et al., 2021).

#### Walking data acquisition

##### Standing task

Subjects were asked to remain upright for at least 3–5 s, and their feet were aligned to obtain baseline data.

##### Walking tasks

Subjects were asked to perform three different walking tasks at a preferred speed on an 8-m footpath: (1) single-task walking (WT): subjects walked at a preferred speed (2) walking + holding a water bottle (WMT) (walking + exercise load as a dual-motion task) (3) walking + computation (WCT): subjects completed consecutive computation tasks of subtracting 7 starting from 100 while walking.

The task sequence was as follows. Subjects completed the baseline gait assessment (WT), and then the order of WMT and WCT dual-task walking was determined by a single-blind random lottery. Each task was performed five times in a row, and the first two to four data were selected as experimental data. If data were missing when processing the data, data from the subsequent experiment were selected to replace them to ensure the integrity of the experimental data.

#### Gait analysis data processing

The BTSFreeEMG300 acquisition system (BTS Engineering, version 2.9.37.0, Italy) was used to analyze the three-dimensional gait data. Firstly, all the gait data collected by the subjects were selected, and at least two heel-strike events and one toe-off event calibrated in the gait cycle were automatically identified by the system. Four gait events were manually checked for accuracy (eRHS: right heel-to-ground, eRTO: right toe-to-ground, eLHS: left heel-to-ground, eLTO: left toe-to-ground). By selecting the “Rep_Gait_Standing” module, we checked whether the label positions of the 22 markers were symmetrical, and by selecting the “Rep_Gait_Consistency” module, we tested the consistency and repeatability of the data. Finally, we generated a module by clicking on “report.”

The spatiotemporal motion parameters for different tasks were obtained, including time parameters (stance phase, swing phase, first double support phase, average velocity, and cadence) and spatial parameters (stride length, step length, and step width). DTC was introduced to calculate the gait-performance difference between single-task gait and dual-task gait and to eliminate the differences in experimental data caused by subjects’ own heterogeneity. The DTC was calculated according to the following: ([single-task gait parameter-dual-task gait parameter]/single-task gait parameter) × 100 (Montero-Odasso et al., 2017).

#### Principal component analysis

Considering the potential correlation between gait variables, PCA method was used to extract the principal features of the 22 spatial-temporal gait variables in order to remove redundancy and locate target parameters. The spatial-temporal data from recruited subjects were imported into SPSS software. For each task paradigm, PCA was performed to identify the potential contribution of spatial-temporal variables, which took into account the changes observed in each task paradigm. For each task paradigm, subjects and variables were arranged in a matrix with gait variables as columns and subjects as rows. A principal component (PC) is represented by varimax rotation to minimize the number of variables with high load on each component factor. PC is retained according to the gravel map describing the individual contribution of each PC to explain the overall variance. The goal was for all retained PCs together to explain ≥ 80% of the total variance. Then Pearson correlation (r) and explicitness value were used to cross-validate the factor load (correlation between original spatial-temporal variables and each PC) and component score coefficient (contribution or weight of spatial-temporal variables to each PC). The contributions of the first two PCs were reported.

### Statistical analysis

After all the data were collected, IBM SPSS statistical software (version 25.0, IBM, Armonk, NY, United States) and GraphPad Prism (La Jolla, CA, United States) were used for statistical analysis and plotting. The data are represented by the mean and standard deviation (SD). In terms of the basic information and spatial-temporal parameters between MCI and NE groups, two independent-sample t-tests were used to compare the differences between the two groups. The repeated-measures analysis of variance was used to analyze the effect of multiple factors (group factors: MCI group and NE group; task factors: WT, WMT, and WCT).

## Results

**FIGURE 1:**
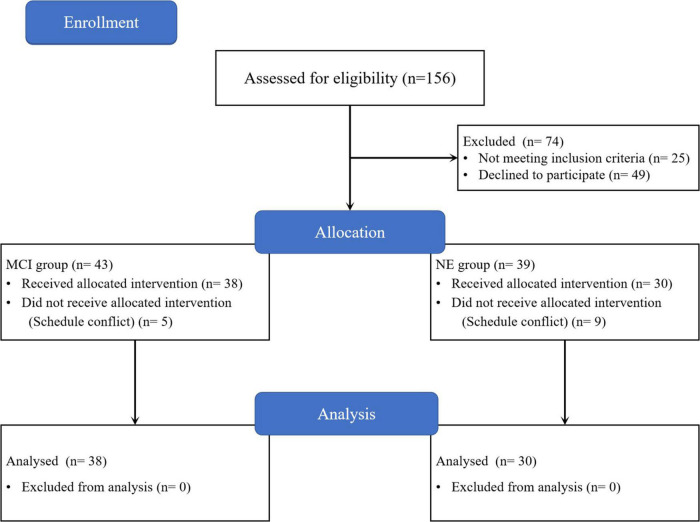
Study flow diagram. NE, normal elder; MCI, mild cognitive impairment.

In this study, 156 subjects were recruited. Of the subjects who met the research conditions, 36 subjects withdrew from the experiment due to low motivation, 13 subjects withdrew because their families did not agree to the experiment, 9 subjects had severe knee osteoarthritis, 16 subjects were beyond the age requirement, and 14 subjects were unable to complete the experiment due to schedule conflicts. During the experiment, no subjects reported any adverse events or results. Finally, 68 subjects completed all the evaluations, including 38 subjects in the MCI group and 30 subjects in the NE group (Figure 1).

The basic information of the subjects can be found in Table 1. There was no significant difference in age, height, weight, BMI, years of education, 10WMT, and TUGT between the two groups (*P* > 0.05). The MoCA score of the MCI group was lower than that of the NE group, and the difference was statistically significant (MCI group: 21.0 ± 1.9; NE group: 26.4 ± 1.4; *p* < 0.001) (Table 1).

**TABLE 1:** Baseline demographic characteristics of the MCI group and NE group.

Characteristics	MCI group	NE group	*P*-value
Age (year)	60.9 ± 7.3	60.1 ± 6.7	0.705
55–64	52.7 ± 2.6 (30)	57.4 ± 2.9 (24)	−
65–74	67.8 ± 3.4 (5)	68.8 ± 2.2 (5)	−
75–85	80.3 ± 4.1 (3)	83.0 ± 0.0 (1)	−
Gender (female/male)	38 (male 7/female 31)	30 (male 2/female 28)	−
Height (cm)	159.7 ± 6.8	159.8 ± 5.4	0.143
Weight (kg)	61.1 ± 9.5	58.2 ± 6.3	0.943
BMI (kg/m^2^)	23.9 ± 3.1	22.8 ± 2.3	0.104
Education (years)	11.0 ± 2.8	11.8 ± 2.6	0.232
MoCA (score)	21.0 ± 1.9	26.4 ± 1.4	<**0.001**
10WMT (s)	5.5 ± 0.7	5.5 ± 0.8	0.91
TUGT (s)	10.9 ± 1.6	10.7 ± 1.7	0.549
MBI (score)	100	100	−

The data are presented as the means ± SD or numbers. P-value less than 0.05 indicate statistically significant differences and are marked in bold.

BMI, body mass index; MoCA, montreal cognitive assessment; TUGT, time up and go test; 10WMT, 10–meter walk test; MBI, modified barthel index.

**FIGURE 2:**
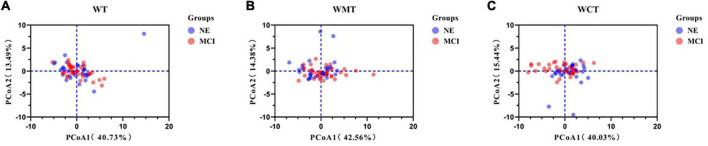
Principal component analysis of spatial-temporal parameters. Component loading plot of spatial-temporal parameters for all 68 patients in three different tasks. **(A)** Walking task; **(B)** walking motor task; **(C)** walking calculate task. WT, walking task; WMT, walking motor task; WCT, walking calculate task; NE, normal elder; MCI, mild cognitive impairment.

All spatial-temporal data for both groups are detailed in the Supplementary Table 1. The PCA results of 22 spatial-temporal parameters of different tasks are shown in Table 2 (WT), Table 3 (WMT), and Table 4 (WCT). Six PCs (PC1–PC6) accounted for 80% of the spatial-temporal changes. Among the three tasks, the parameters with the highest weight proportion of PC1 were cadence (fqCADENCE, fqRCADENCE, fqLCADENCE) (Tables 2–4). At the same time, PC2 of WT and WMT had the highest proportion weight for gait substage parameters (swing and stance), and PC2 of WCT had the highest proportion weight for spatial parameters (LSTRIDE% height, RSTRIDE% height). Figure 2 shows the data points of the three walking tasks (68 * 22 * 3 * 1496 measurements in the three tasks from 68 subjects) projected onto a two-dimensional plane based on two spatiotemporal variations.

**TABLE 2:** Results of principal component analysis of WT.

Parameters	Component					
	
	PC 1	PC 2	PC 3	PC 4	PC 5	PC 6
velseqRHSTRIDE	0.473	0.744	0.199	0.075	−0.021	0.224
velseqLHSTRIDE	0.656	0.501	0.43	0.066	0.035	−0.097
velseqRHSWING	0.759	0.107	0.544	0.021	0.002	0.235
velseqLHSWING	0.732	0.196	0.533	0.113	−0.058	0
fqRCADENCE	**0.922** ^a^	−0.029	0.216	0.002	−0.018	0.088
fqLCADENCE	**0.868^a^**	0.032	0.294	−0.01	−0.003	−0.233
fqCADENCE	**0.906^a^**	0.357	0.039	−0.008	−0.014	−0.051
sRSTANCE	−0.029	−**0.919^a^**	0.186	−0.121	0.027	0.143
sRSWING	0.178	**0.812a**	0.154	0.205	−0.055	−0.081
sLDBLSTANCE	−0.506	−0.185	0.142	−0.03	0.108	0.459
sLSTANCE	−0.043	−**0.854^a^**	0.019	0.166	−0.244	−0.062
sLSWING	0.186	0.731	0.389	0.011	0.113	0.153
sRDBLSTANCE	−0.289	−0.551	−0.517	0.077	0.043	0.163
RGDI	−0.068	−0.177	−0.029	−**0.892^a^**	−0.2	−0.059
sRGPS	−0.023	−0.045	0.002	**0.933^a^**	0.091	−0.087
LGDI	0.041	−0.138	0.085	−0.169	−**0.915^a^**	−0.059
sLGPS	−0.02	−0.002	0.135	0.114	**0.916^a^**	0.035
sRSINGSTANCE	0.122	0.507	0.293	0.028	0.107	**0.731^a^**
sLSINGSTANCE	0.222	0.521	0.37	0.12	−0.008	−**0.637^a^**
velMEAN%height/s	0.71	0.37	0.506	−0.014	0.02	−0.07
LSTRIDE%height	0.299	0.082	**0.91^a^**	0.026	0.051	0.129
RSTRIDE%height	0.337	0.047	**0.911^a^**	0.011	0.039	0.017

The parameters that obtained membership to each component (>0.500) are in bold and with the superscript symbol “a”.

velseqRHSTRIDE, right heel stride velocity; velseqLHSTRIDE, left heel stride velocity; velseqRHSWING, right heel swing velocity; velseqLHSWING, left heel swing velocity; fqRCADENCE, the cadence of right foot; fqLCADENCE, the cadence of left foot; fqCADENCE, the cadence of double feet; sRSTANCE, right stance phase; sRSWING, right swing phase; sRDBLSTANCE, right double support phase; sLSTANCE, left stance phase; sLSWING, left swing phase; sLDBLSTANCE, left double support phase; RGDI, right gait deviation index; LGDI, left gait deviation index; sRGPS, right gait profile score; sLGPS, left gait profile score; velMEAN%height/s, mean velocity in relation to the height of the subject; RSTRIDE%height, length of the right stride in relation to the height of the subject; LSTRIDE%height, length of the left stride in relation to the height of the subject.

**TABLE 3:** Results of principal component analysis of WMT.

Parameters	Component					
	
	PC 1	PC 2	PC 3	PC 4	PC 5	PC 6
velseqRHSTRIDE	0.473	0.744	0.199	0.075	−0.021	0.224
velseqLHSTRIDE	0.656	0.501	0.43	0.066	0.035	−0.097
velseqRHSWING	0.759	0.107	0.544	0.021	0.002	0.235
velseqLHSWING	0.732	0.196	0.533	0.113	−0.058	0
fqRCADENCE	**0.922^a^**	−0.029	0.216	0.002	−0.018	0.088
fqLCADENCE	**0.868^a^**	0.032	0.294	−0.01	−0.003	−0.233
fqCADENCE	**0.906^a^**	0.357	0.039	−0.008	−0.014	−0.051
sRSTANCE	−0.029	−**0.919^a^**	0.186	−0.121	0.027	0.143
sRSWING	0.178	**0.812^a^**	0.154	0.205	−0.055	−0.081
sLDBLSTANCE	−0.506	−0.185	0.142	−0.03	0.108	0.459
sLSTANCE	−0.043	−**0.854^a^**	0.019	0.166	−0.244	−0.062
sLSWING	0.186	0.731	0.389	0.011	0.113	0.153
sRDBLSTANCE	−0.289	−0.551	−0.517	0.077	0.043	0.163
RGDI	−0.068	−0.177	−0.029	−**0.892^a^**	−0.2	−0.059
sRGPS	−0.023	−0.045	0.002	**0.933^a^**	0.091	−0.087
LGDI	0.041	−0.138	0.085	−0.169	−**0.915^a^**	−0.059
sLGPS	−0.02	−0.002	0.135	0.114	**0.916^a^**	0.035
sRSINGSTANCE	0.122	0.507	0.293	0.028	0.107	**0.731^a^**
sLSINGSTANCE	0.222	0.521	0.37	0.12	−0.008	−**0.637^a^**
velMEAN%height/s	0.71	0.37	0.506	−0.014	0.02	−0.07
LSTRIDE%height	0.299	0.082	**0.91^a^**	0.026	0.051	0.129
RSTRIDE%height	0.337	0.047	**0.911^a^**	0.011	0.039	0.017

The parameters that obtained membership to each component (>0.500) are in bold and with the superscript symbol “a”.

velseqRHSTRIDE, right heel stride velocity; velseqLHSTRIDE, left heel stride velocity; velseqRHSWING, right heel swing velocity; velseqLHSWING, left heel swing velocity; fqRCADENCE, the cadence of right foot; fqLCADENCE, the cadence of left foot; fqCADENCE, the cadence of double feet; sRSTANCE, right stance phase; sRSWING, right swing phase; sRDBLSTANCE, right double support phase; sLSTANCE, left stance phase; sLSWING, left swing phase; sLDBLSTANCE, left double support phase; RGDI, right gait deviation index; LGDI, left gait deviation index; sRGPS, right gait profile score; sLGPS, left gait profile score; velMEAN%height/s, mean velocity in relation to the height of the subject; RSTRIDE%height, length of the right stride in relation to the height of the subject; LSTRIDE%height, length of the left stride in relation to the height of the subject.

**TABLE 4:** Results of principal component analysis of WCT.

Parameters	Component					
	PC 1	PC 2	PC 3	PC 4	PC 5	PC 6
velseqRHSTRIDE	0.413	0.32	0.613	0.481	0.016	−0.036
velseqLHSTRIDE	0.388	0.365	0.639	0.433	−0.024	−0.023
velseqRHSWING	0.718	0.592	−0.028	0.287	0.026	−0.024
velseqLHSWING	0.659	0.667	0.107	0.188	−0.015	0.015
fqRCADENCE	**0.95^a^**	0.143	0.03	0.224	−0.02	−0.046
fqLCADENCE	**0.966^a^**	0.169	0.066	0.035	−0.05	−0.024
fqCADENCE	**0.97^a^**	0.071	0.151	−0.008	−0.042	−0.036
sRSTANCE	0.035	0.166	−**0.948^a^**	−0.023	0.011	0.036
sRSWING	0.054	0.358	0.638	0.481	0.024	−0.051
sLDBLSTANCE	−0.062	−0.33	−0.236	0.392	0.533	0.173
sLSTANCE	−0.036	0.192	−0.86	−0.225	0.007	0.063
sLSWING	0.16	0.265	0.494	**0.747^a^**	0.025	−0.094
sRDBLSTANCE	−0.112	−0.199	−0.07	−0.363	0.094	0.256
RGDI	0.067	−0.149	−0.245	0.067	−**0.838^a^**	0.285
sRGPS	−0.008	−0.036	−0.122	−0.01	**0.933^a^**	−0.113
LGDI	0.038	−0.003	−0.191	−0.083	−0.112	**0.93^a^**
sLGPS	0.095	−0.037	−0.106	0.125	0.179	−**0.892^a^**
sRSINGSTANCE	0.183	0.247	0.199	**0.861^a^**	0.081	−0.121
sLSINGSTANCE	0.129	0.404	0.707	−0.02	−0.038	0
velMEAN%height/s	0.67	0.664	0.161	0.217	−0.036	−0.023
LSTRIDE%height	0.178	**0.928^a^**	0.01	0.239	−0.008	−0.008
RSTRIDE%height	0.221	**0.917^a^**	0.055	0.203	0.002	0.007

The parameters that obtained membership to each component (>0.500) are in bold and with the superscript symbol “a”

velseqRHSTRIDE, right heel stride velocity; velseqLHSTRIDE, left heel stride velocity; velseqRHSWING, right heel swing velocity; velseqLHSWING, left heel swing velocity; fqRCADENCE, the cadence of right foot; fqLCADENCE, the cadence of left foot; fqCADENCE, the cadence of double feet; sRSTANCE, right stance phase; sRSWING, right swing phase; sRDBLSTANCE, right double support phase; sLSTANCE, left stance phase; sLSWING, left swing phase; sLDBLSTANCE, left double support phase; RGDI, right gait deviation index; LGDI, left gait deviation index; sRGPS, right gait profile score; sLGPS, left gait profile score; velMEAN%height/s, mean velocity in relation to the height of the subject; RSTRIDE%height, length of the right stride in relation to the height of the subject; LSTRIDE%height, length of the left stride in relation to the height of the subject.

Table 5 shows results of the repeated-measures analysis of variance on group factor (MCI group and NE group) and task factors (WT, WMT, and WCT) for fqRCADENCE, fqLCADENCE, fqCADENCE. The raw data of fqCADENCE and the DTC of the three CADENCE parameters were significantly different among tasks and groups (group factor: Raw Data_fqCADENCE_: *F* = 6.310, *P* = 0.013; DTC_fqRCADENCE_: *F* = 22.021, *P* < 0.001; DTC_fqLCADENCE_: *F* = 9.121, *P* = 0.003; DTC_fqCADENCE_: *F* = 17.054, *P* < 0.001; task factor: Raw Data_fqCADENCE_: *F* = 6.226, *P* = 0.002; DTC_fqRCADENCE_: *F* = 23.616, *P* < 0.001; DTC_fqLCADENCE_: *F* = 8.957, *P* = 0.003; DTC_fqCADENCE_: *F* = 19.537, *P* < 0.001). The raw data of fqRCADENCE and fqLCADENCE just presented significant difference among three walking tasks (Raw Data_fqRCADENCE_: *F* = 4.220, *P* = 0.016; Raw Data_fqLCADENCE_: *F* = 4.256, *P* = 0.015).

**TABLE 5:** Results of the repeated-measures analysis of variance on group and task for fqRCADENCE, fqLCADENCE, fqCADENCE.

	Main effect		Main effect		Interaction effect	
	(Group)		(Task)			
			
	*F*-value	*P*-value	*F*-value	*P*-value	*F*-value	*P*-value
**Raw data (steps/min)**						
fqRCADENCE	3.044	0.083	4.220	**0.016**	1.220	0.297
fqLCADENCE	3.842	0.051	4.256	**0.015**	0.839	0.434
fqCADENCE	6.310	**0.013**	6.226	**0.002**	1.401	0.249
**DTC (%)**						
fqRCADENCE	22.021	**<0.001**	23.616	**<0.001**	1.117	0.292
fqLCADENCE	9.121	**0.003**	8.957	**0.003**	0.603	0.439
fqCADENCE	17.054	**<0.001**	19.537	**<0.001**	1.055	0.306

DTC = 100 * (single-task gait parameters − dual-task gait parameters)/single-task gait parameters; *P*-value less than 0.05 indicate statistically significant differences and are marked in bold.

fqRCADENCE, the cadence of right foot; fqLCADENCE, the cadence of left foot; fqCADENCE, the cadence of double feet.

Table 6 shows the differences in fqCADENCE among single and dual walking tasks in the MCI group and NE group respectively. In the MCI group, the fqCADENCE in WCT was significantly smaller than that in WT (Mean difference = 4.442 ± 1.144 steps/min, *P* = 0.001). Whereas, in the NE group, the fqCADENCE in WMT was significantly smaller than that in WT (Mean difference = −6.924 ± 1.147 steps/min, *P* < 0.001). Moreover, the fqCADENCE between MCI group and NE group in WMT were significantly larger than those in WCT (MCI group: Mean difference = 6.537 ± 1.153 steps/min, *P* < 0.001; NE group: Mean difference = 5.790 ± 1.298 steps/min, *P* < 0.001). The DTC_fqRCADENCE_ in WMT in both groups were significantly smaller than those in WCT (MCI group: Mean difference = −5.970 ± 0.868%, *P* < 0.001; NE group: Mean difference = −3.718 ± 0.977%, *P* < 0.001).

**TABLE 6:** Paired comparison of fqCADENCE parameters (raw date and DTC) in different tasks respectively.

	MCI group		NE group	
		
	Mean difference (Mean ± SE)	*P*-value	Mean difference (Mean ± SE)	*P*-value
**Raw data (steps/min)**				
WT vs. WMT	−2.095 ± 1.019	0.132	−6.924 ± 1.147	**<0.001**
WT vs. WCT	4.442 ± 1.144	**0.001**	−1.134 ± 1.288	1.000
WMT vs. WCT	6.537 ± 1.153	**<0.001**	5.790 ± 1.298	**<0.001**
**DTC (%)**				
WMT vs. WCT	−5.970 ± 0.868	**<0.001**	−3.718 ± 0.977	**<0.001**

DTC = 100 * (single-task gait parameters − dual-task gait parameters)/single-task gait parameters; *P*-value less than 0.05 indicate statistically significant differences and are marked in bold.

SE, standard error; WT, walking task; WMT, walking motor task; WCT, walking calculate task; DTC, Dual task cost.

Table 7 shows the difference in fqCADENCE between the MCI group and the NE group in different single and dual walking tasks respectively. The fqCADENCE of the MCI group in WMT and WCT were both significantly smaller than those of the NE group (WMT: Mean difference = −5.069 ± 2.326 steps/min, *P* = 0.033; WCT: Mean difference = −5.816 ± 2.684 steps/min, *P* = 0.034). Moreover, the DTC_fqRCDENCE_ of the MCI group both in WMT and WCT were significantly smaller than those of the NE group (WMT: Mean difference = 3.400 ± 1.390%, *P* = 0.017; WCT: Mean difference = 5.652 ± 1.694%, *P* = 0.001).

**TABLE 7:** Paired comparison of fqCADENCE parameters (raw date and DTC) between MCI group and NE group.

	Mean difference (Mean ± SE)	*P*-value
**Raw data (steps/min)**		
WT	−0.240 ± 2.645	0.928
WMT	−5.069 ± 2.326	**0.033**
WCT	−5.816 ± 2.684	**0.034**
**DTC (%)**		
WMT	3.400 ± 1.390	**0.017**
WCT	5.652 ± 1.694	**0.001**

DTC = 100 * (single-task gait parameters − dual-task gait parameters)/single-task gait parameters; *P*-value less than 0.05 indicate statistically significant differences and are marked in bold.

SE, standard error; WT, walking task; WMT, walking motor task; WCT, walking calculate task; DTC, Dual task cost.

## Discussion

In this study, the difference of gait parameters between MCI patients and healthy elderly people in single and dual tasks was explored by using dual-task three-dimensional gait analysis. The aim was to explore the biomechanical targets of MCI patients and optimize the clinical quantitative evaluation and identification methods for MCI patients. The results of principal component analysis showed that the cadence parameters (fqRCADENCE, fqLCADENCE, and fqCADENCE) presented the highest weight in distinguishing MCI group and NE group. Furthermore, the repeated-measures analysis of variance results showed that the significant advantages of three DTCcadence parameters in distinguishing MCI group and NE group, while only raw date of the fqCADENCE parameter presented significant differences between groups. At the same time, the significant differences between the two groups under different dual tasks were found in both in raw date and DTC, indicating that dual task paradigm of gait analysis could be used to distinguish MCI group and NE group. The more significant difference between two groups were under the DTC than raw data, Moreover, the more significant difference was found in WCT than in WMT.

The effectiveness of dual-task gait analysis in identifying MCI has been confirmed in previous studies. Doi et al. (2014) compared the gait velocity of MCI patients between a single task and dual task (walking while counting backward from 100) and found that the working memory ability of MCI patients in the dual task significantly affected their gait velocity. There was no significant difference in the single task. Furthermore, Montero-Odasso et al. found that the DTC parameters of walking speed in the reciprocal (counting backward) dual-task walking test could be used to predict the progress of dementia in MCI patients by constructing a prediction model. They confirmed the potential of a dual-task gait test for clinical cognitive function assessment (Montero-Odasso et al., 2017). This study also found that the DTC value of step frequency of MCI patients in dual tasks (walking while counting down from 100 by 7) was greater than that of the normal elderly subjects, which was similar to the result of other studies. The DTC value of cadence can be used as an important biomechanical indicator to identify MCI, which once again verifies the positive role of dual-task gait analysis in clinical evaluation and diagnosis of MCI.

There are many biomechanical indicators available from gait analysis, but accurate determination of target indicators is of great significance for the clinical application and promotion of MCI diagnosis based on this method. Therefore, we used PCA to reduce the dimensions of the spatial-temporal parameters in three walking tasks and selected cadence as the index with the largest proportion weight for in-depth intra-group comparison. Cadence refers to the frequency of legs switching support points when running or walking. The results of PCA showed that cadence had the largest weight in distinguishing the MCI group and the NE group. The possible reasons are as follows. First, when walking at a preferred velocity, factors such as height and leg length may lead to inconsistent individual strides, resulting in inconsistent paces. Therefore, when comparing the two groups, simply considering the pace may be biased. When studying the cognitive function training effect of MCI patients, Liao et al. (2019) found that DTC_cadence_ results were statistically significant, while DTC_velocity_ was not, which was similar to the results of this study. Second, steps/min is the unit of step cadence, and m/s is the unit of gait velocity. Cadence is measured per minute, and the gait velocity is measured per second. Thus, in the statistical analysis, the cadence parameter makes the differences accumulate compared with the gait velocity, which is more conducive to identifying the cognitive-function difference between MCI and NE groups. Therefore, cadence may be more likely to be an important biomechanical marker to identify MCI gait decline. Third, in previous studies, velocity was often used as an important result indicator in the analysis of dual-task gait. This may be due to the fact that the gait velocity parameters are more easily obtained in clinical evaluation, such as the commonly used 10-meter walking test and TUGT test. The gait velocity parameters can be obtained with only timing tools, while the cadence parameters are difficult to obtain in non-instrumental evaluation. In this study, a three-dimensional gait analysis system was used for the dual-task gait analysis, and the gait parameters such as cadence and gait velocity can be obtained easily. The results showed that the cadence of the MCI group and the NE group decreased in the dual task, and the decrease of the MCI group was more obvious than that of the NE group. This result can be used to confirm that MCI can lead to a decrease of dual-task gait performance, which can be used to identify MCI patients and new ideas for MCI gait research. The results indicated that DTC parameters might be more sensitive than raw data in distinguishing MCI group and NE group. DTC parameters can be used for quantification of dual tasks and comparison between different dual-task paradigms. At the same time, DTC can remove gait physiological differences between different individuals by calculating the difference between single and dual tasks, as well as reduce the impact of heterogeneity on experimental design (Bayot et al., 2018). Due to these advantages of DTC, DTC_cadence_ is more effective than raw cadence in distinguishing MCI and healthy subjects. Ying et al. also studied the effect of VR-based physical and cognitive training on the performance of dual-task gait in MCI patients. They found that DTC_cadence_ was improved, while the original data of step frequency had no statistical difference. Thus, promptly obtained DTC parameters have advantages for clinical assessment and diagnosis of MCI. The significant differences between the two groups under different dual tasks were found both in raw date and DTC. Moreover, the more significant difference was found in WCT than in WMT under the DTC. Our results suggested that both the motor-cognitive dual task and dual motor task in this study could be used to distinguish MCI patients from healthy elderly, however, the motor-cognitive dual task presented more advantageous in the diagnosis of MCI population. The results are also consistent with the pathological characteristics of MCI—that is the ADL of MCI patients is partially retained, while the cognitive function is impaired.

Furthermore, the dual-motor task WMT designed in this study involves holding a water bottle and walking, which can be regarded as a single motor task whether (holding a water bottle or walking) (McIsaac et al., 2015). The demand for cognitive brain function resources of the subjects is not large, and it is not easy to occupy significant cognitive resources compared to the single walking task. However, the subjects in WCT with continuous subtraction by 7 needed more cognitive brain function resources, which is more challenging for MCI patients (Tsang et al., 2022). Therefore, WCT is more likely to identify MCI patients than WMT. Thus, there are many types of dual tasks in clinical evaluation, and it is possible to choose motor-cognitive tasks with higher sensitivity for MCI recognition than dual motor tasks.

### Limitations

This study has the following limitations. Most of the subjects were female, and few were male. However, due to the high prevalence of women among MCI patients, the gender ratio of subjects was in line with epidemiological characteristics (Jia et al., 2020; Shi et al., 2022). In subsequent studies, we will include more male subjects to reduce the impact of gender on the experimental results. The key spatial-temporal parameters for MCI recognition under dual-task gait were discussed, and future research should further explore the influence of joint angle on MCI recognition. In the future, we will continue follow-up on this this study by obtaining data before and after healthy subjects develop MCI, which is of great significance for further clarifying the recognition of MCI through gait parameters. Further studies should also improve the efficiency of clinical assessment and diagnosis of MCI by constructing a clinical prediction model through the dual-task gait parameters of MCI patients.

## Conclusion

This study examined dual-task three-dimensional gait analysis methods, and the key spatial-temporal parameters for the early diagnosis of MCI were screened through PCA. The results showed that the DTC_cadence_ based on the cognitive-motor dual-task gait analysis has potential application value for the early recognition of MCI. The results could provide theoretical support for improving the clinical diagnosis of MCI.

## Data availability statement

The raw data supporting the conclusions of this article will be made available by the corresponding authors, upon reasonable request.

## Ethics statement

The studies involving human participants were reviewed and approved by the Fifth Affiliated Hospital of Guangzhou Medical University. The patients/participants provided their written informed consent to participate in this study.

## Author contributions

QL, HO, and YZ designed the study. QL, YZ, and SL drafted the manuscript. YZ, SL, JL, and YJ performed data analysis. JL, HC, YJ, BZ, AY, DH, QYL, HL, SC, FX, and HO collected the data. YJ, BZ, QYL, and HL wrote sections of the manuscript. QL and HO approved the final version of the manuscript. All authors contributed to the manuscript revision, read, and approved the submitted version.

## Footnotes

1
https://www.chictr.org.cn
