# Supplementary material for: Effects of motor-cognitive interaction based on dual-task gait analysis recognition in middle age to aging people with normal cognition and mild cognitive impairment
Source: Front Aging Neurosci. 2022 Oct 4;14:969822. doi: 10.3389/fnagi.2022.969822 (PMC9577255; doi:10.3389/fnagi.2022.969822)
Supplement: Supplementary file 1 [file Table_1.docx]

**Supplementary Table 1 The mean value and standard deviation of** **spatial and temporal parameters in MCI group and NE group under WT, WMT and WCT.**

| **Parameter** |  | **WT** | | | | | | |  | **WMT** | | | | | | |  | **WCT** | | | | | | |
| --- | --- | --- | --- | --- | --- | --- | --- | --- | --- | --- | --- | --- | --- | --- | --- | --- | --- | --- | --- | --- | --- | --- | --- | --- |
|  |  | **MCI** | | |  | **NE** | | |  | **MCI** | | |  | **NE** | | |  | **MCI** | | |  | **NE** | | |
| **velseqRHSTRIDE** |  | 1.065 | ± | 0.137 |  | 1.102 | ± | 0.136 |  | 1.104 | ± | 0.152 |  | 1.196 | ± | 0.159 |  | 1.002 | ± | 0.181 |  | 1.105 | ± | 0.166 |
| **velseqLHSTRIDE** |  | 1.068 | ± | 0.136 |  | 1.123 | ± | 0.171 |  | 1.110 | ± | 0.163 |  | 1.175 | ± | 0.128 |  | 0.999 | ± | 0.176 |  | 1.104 | ± | 0.155 |
| **velseqRHSWING** |  | 2.461 | ± | 0.266 |  | 2.445 | ± | 0.331 |  | 2.522 | ± | 0.300 |  | 2.566 | ± | 0.274 |  | 2.344 | ± | 0.341 |  | 2.395 | ± | 0.315 |
| **velseqLHSWING** |  | 2.443 | ± | 0.268 |  | 2.451 | ± | 0.312 |  | 2.495 | ± | 0.279 |  | 2.614 | ± | 0.274 |  | 2.323 | ± | 0.336 |  | 2.403 | ± | 0.275 |
| **fqRCADENCE** |  | 55.558 | ± | 4.147 |  | 55.394 | ± | 6.275 |  | 56.463 | ± | 4.647 |  | 57.860 | ± | 6.465 |  | 53.132 | ± | 4.914 |  | 55.863 | ± | 5.963 |
| **fqLCADENCE** |  | 55.300 | ± | 4.004 |  | 55.522 | ± | 6.969 |  | 56.532 | ± | 5.195 |  | 58.253 | ± | 6.626 |  | 53.284 | ± | 5.045 |  | 56.007 | ± | 6.032 |
| **fqCADENCE** |  | 110.926 | ± | 7.977 |  | 111.166 | ± | 13.632 |  | 113.021 | ± | 9.419 |  | 118.090 | ± | 9.652 |  | 106.484 | ± | 9.916 |  | 112.300 | ± | 12.225 |
| **sRSTANCE** |  | 60.297 | ± | 1.534 |  | 124.821 | ± | 362.131 |  | 60.150 | ± | 1.391 |  | 58.415 | ± | 3.008 |  | 61.053 | ± | 1.695 |  | 59.005 | ± | 3.996 |
| **sRSWING** |  | 39.726 | ± | 1.451 |  | 40.434 | ± | 1.209 |  | 39.850 | ± | 1.391 |  | 41.152 | ± | 2.075 |  | 38.947 | ± | 1.695 |  | 40.220 | ± | 1.823 |
| **sLDBLSTANCE** |  | 11.023 | ± | 5.436 |  | 11.296 | ± | 7.050 |  | 10.012 | ± | 1.582 |  | 10.444 | ± | 5.590 |  | 10.799 | ± | 2.054 |  | 10.958 | ± | 5.389 |
| **sLSTANCE** |  | 59.998 | ± | 1.708 |  | 59.387 | ± | 4.245 |  | 59.342 | ± | 2.392 |  | 58.696 | ± | 2.958 |  | 60.794 | ± | 1.842 |  | 58.810 | ± | 3.927 |
| **sLSWING** |  | 40.002 | ± | 1.708 |  | 40.189 | ± | 3.541 |  | 40.326 | ± | 2.306 |  | 40.863 | ± | 1.986 |  | 39.178 | ± | 1.826 |  | 40.431 | ± | 1.968 |
| **sRDBLSTANCE** |  | 10.968 | ± | 1.542 |  | 10.929 | ± | 5.693 |  | 10.336 | ± | 1.384 |  | 9.642 | ± | 1.368 |  | 12.098 | ± | 5.083 |  | 10.727 | ± | 1.557 |
| **RGDI** |  | 83.640 | ± | 11.447 |  | 82.757 | ± | 12.652 |  | 83.414 | ± | 11.403 |  | 82.479 | ± | 11.051 |  | 83.296 | ± | 10.668 |  | 81.842 | ± | 10.744 |
| **sRGPS** |  | 911.193 | ± | 304.123 |  | 843.874 | ± | 258.126 |  | 894.618 | ± | 237.248 |  | 898.699 | ± | 279.230 |  | 876.265 | ± | 236.921 |  | 883.549 | ± | 268.316 |
| **LGDI** |  | 87.590 | ± | 13.275 |  | 84.754 | ± | 14.564 |  | 86.984 | ± | 13.161 |  | 84.898 | ± | 12.030 |  | 87.999 | ± | 14.927 |  | 84.987 | ± | 12.691 |
| **sLGPS** |  | 741.038 | ± | 261.798 |  | 794.043 | ± | 263.053 |  | 749.652 | ± | 270.750 |  | 836.291 | ± | 284.913 |  | 747.508 | ± | 263.796 |  | 808.141 | ± | 275.474 |
| **sRSINGSTANCE** |  | 40.376 | ± | 2.323 |  | 39.916 | ± | 3.803 |  | 40.248 | ± | 2.067 |  | 40.671 | ± | 2.777 |  | 39.108 | ± | 2.091 |  | 40.172 | ± | 2.488 |
| **sLSINGSTANCE** |  | 39.781 | ± | 2.148 |  | 40.239 | ± | 3.151 |  | 40.028 | ± | 2.651 |  | 41.225 | ± | 2.021 |  | 39.095 | ± | 2.040 |  | 40.326 | ± | 1.472 |
| **velMEAN %height/s** |  | 67.927 | ± | 9.410 |  | 67.170 | ± | 12.175 |  | 70.014 | ± | 10.636 |  | 73.300 | ± | 9.193 |  | 63.564 | ± | 11.566 |  | 66.371 | ± | 9.763 |
| **LSTRIDE %height** |  | 72.787 | ± | 8.630 |  | 71.369 | ± | 8.992 |  | 74.027 | ± | 7.245 |  | 73.683 | ± | 7.697 |  | 70.977 | ± | 8.764 |  | 70.171 | ± | 8.257 |
| **RSTRIDE %height** |  | 72.549 | ± | 8.691 |  | 71.673 | ± | 8.966 |  | 74.035 | ± | 7.221 |  | 73.499 | ± | 7.779 |  | 71.158 | ± | 8.669 |  | 70.518 | ± | 8.230 |

Abbreviations: WT, walking task; WMT, walking motor task; WCT, walking calculate task; NE, normal elder; MCI, mild cognitive impairment; velseqRHSTRIDE, right heel stride velocity; velseqLHSTRIDE, left heel stride velocity; velseqRHSWING, right heel swing velocity; velseqLHSWING, left heel swing velocity; fqRCADENCE, the cadence of right foot; fqLCADENCE, the cadence of left foot; fqCADENCE, the cadence of double feet; sRSTANCE, right stance phase; sRSWING, right swing phase; sRDBLSTANCE, right double support phase; sLSTANCE, left stance phase; sLSWING, left swing phase; sLDBLSTANCE, left double support phase; RGDI, right gait deviation index; LGDI, left gait deviation index; sRGPS, right gait profile score; sLGPS, left gait profile score; velMEAN %height/s, mean velocity in relation to the height of the subject; RSTRIDE %height, length of the right stride in relation to the height of the subject;LSTRIDE %height, length of the left stride in relation to the height of the subject.
